# Control of standing balance at leaning postures with functional neuromuscular stimulation following spinal cord injury

**DOI:** 10.1007/s11517-017-1687-x

**Published:** 2017-07-24

**Authors:** Musa L. Audu, Brooke M. Odle, Ronald J. Triolo

**Affiliations:** 1 Department of Orthopaedics, Case Western Reserve University, Cleveland, OH USA; 2 Department of Biomedical Engineering, Case Western Reserve University, Cleveland, OH USA; 3 Advanced Platform Technology Center, Cleveland Louis Stokes Veterans Affairs Medical Center, 10701 East Boulevard, Cleveland, OH 44106 USA; 4 Motion Study Laboratory, C15, VA Medical Center, Cleveland, OH 44106 USA

**Keywords:** Spinal cord injury, Standing balance, Posture controller, Functional neuromuscular stimulation, Feedback control of standing postures

## Abstract

This study systematically explored the potential of applying feedback control of functional neuromuscular stimulation (FNS) for stabilizing various erect and leaning standing postures after spinal cord injury (SCI). Perturbations ranging from 2 to 6% body weight were applied to two subjects with motor complete thoracic level SCI who were proficient at standing with implanted multichannel neural stimulators to activate the ankle, knee, hip and trunk muscles. The subjects stood with four different postures: erect, forward, forward-right and forward-left. Repeatable and controlled perturbations were applied in the forward, backward, rightward and leftward directions by linear actuators pulling on ropes attached to the subjects via a belt worn just above the waist. Upper extremity (UE) forces exerted on a stationary walker were measured with load cells attached to the handles. A feedback controller based on center of pressure (CoP) varied the stimulation levels to the otherwise paralyzed muscles so as to resist the effects of the perturbations. The effect of the feedback controller was compared to the case where only open-loop baseline stimulation was applied. This was done in terms of: (a) maximum resultant UE force exerted by the subjects on the walker, (b) maximum resultant CoP overshoot and (c) CoP root-mean-square deviation (RMSD). Feedback control resulted in significant reductions in the mean values of the majority of outcome values compared to baseline open-loop stimulation. Maximum resultant UE force was reduced by as much as 50% in one of the postures for one of the subjects. RMSD and maximum CoPs were reduced by as much as 75 and 70%, respectively, with feedback control. These results indicate that feedback control can be used to reject destabilizing disturbances in individuals with SCI using FNS not only for erect postures but also for leaning postures typically adopted during reaching while attempting various activities of daily living.

## Introduction

Neural prostheses employing functional neuromuscular stimulation (FNS) can restore erect standing to individuals with thoracic level, motor complete spinal cord injury (SCI). This is often achieved by maximally activating the hip, knee and trunk extensor muscles by exciting the intact peripheral motor nerves with small stimulating currents [10, 17, 18, 26]. Many activities of daily living, however, require standing at non-erect postures that shift the body anteriorly or medial-laterally away from vertical. Such functional reaching movements are achieved by leaning with the whole body in such a way that relocates the center of mass within the base of support (BoS) without moving the feet [12]. For individuals with SCI using currently available standing neural prostheses, such a maneuver can only be achieved by exerting effort on a support device with the upper extremities (UEs) to overcome the constant maximal activation of the muscles required to achieve the nominal upright posture with the head, trunk, pelvis and legs aligned vertically. The overall effect is to induce rapid fatigue in both the muscles and the UEs which in turn leads to reductions in standing times. Furthermore, reliance on the UEs compromises the ability to reach and manipulate objects in the environment while standing with the neuroprosthesis. The overall goal is to afford system users the ability to assume task-dependent forward and side leaning postures while simultaneously reducing the UE forces required to maintain balance and recover from perturbations. Another implicit effect of leaning away from an erect posture is the movement of the center of pressure (CoP) toward the edge of the BoS which increases the potential of the system to be destabilized by internal or external disturbances [35].

Several studies had extensively investigated feedback (closed-loop) control systems for standing balance with FNS. A number of these were based on joint angles as feedback signals and many were implemented on an individual joint basis such as the knees [16], hips [9], and ankles [22]. These studies showed measures of improvement in disturbance response but effectively constrained the standing system to single planes of movement. Recent interest has focused on using feedback control to (1) reduce the amount of muscle activity required to maintain an erect stance and (2) assist neural prosthesis users to shift posture or maintain balance in the presence of internally generated or externally applied perturbations [19, 23, 25, 34]. With feedback control, it should be possible to recruit muscles at or below their maximal capacities with corrections being made to adjust for deviations from desired behavior. Abbas and Chizeck [1] examined the application of feedback control for maintaining standing balance in the coronal plane using hip ab/adduction angle measured with a liquid–metal strain gage as the feedback signal. In their experiments, the subjects were perturbed by forces of varying magnitude while standing in an erect posture aided by braces to minimize motion in the sagittal plane. The feedback control system resulted in a 41% reduction in root-mean-squared (rms) error (defined as the deviation from the desired hip ab/adduction angle); a 52% reduction in steady-state error, and a 22% reduction in hip compliance. In [14], Gollee et al. designed and deployed a robust FNS feedback controller for maintaining standing balance in the sagittal plane. In their experiments with SCI subjects, the knees, hips and trunk were locked so that movement at the ankle joints alone was allowed. With such constraints, hands-free experiments were made feasible. Disturbances to erect stance were applied by pushing or pulling on the subject. The disturbances were quantified by computing the resulting moment at the ankle. When the disturbances were of the order of ±20 N-m, standing balance could not be maintained while for lower moments (±15 N-m) the feedback controller successfully maintained standing balance. In another series of experiments, SCI subjects using FNS and feedback control systems based on center of mass acceleration (CoMA) were subjected to perturbations internally generated by voluntary arm movements or externally applied at various intensities, directions and locations by linear actuators while in an erect standing posture [28, 29]. Averaged over all conditions, the CoMA feedback control systems resulted in reductions of the order of 33% body weight (BW) in the forces produced by the arms to maintain an upright posture in response to external perturbations and as much as 27% of BW in response to internally generated perturbations. Peak values in UE force reductions approached 50% in some cases. These studies demonstrated the usefulness of feedback control in maintaining a neutral, erect standing posture with FNS alone. To enhance the utility of FNS for undertaking activities of daily living (ADL) while standing, it is imperative to enable neural prosthesis users lean away from the erect posture such as in the forward or sideways directions. Advanced control systems for standing neuroprostheses need to assist with maintaining non-erect postures to allow users to prepare to perform ADL tasks that require deviation from vertical. Preliminary studies in computer simulation of standing balance with the user at several forward-leaning postures have predicted that posture can be maintained by appropriately modulating stimulation via feedback control in a way that can reduce UE effort over continuous open-loop activation [31]. This has, however, not yet been demonstrated experimentally in subjects with SCI using FNS. Our objectives in this study were to: (1) design and clinically implement a closed-loop feedback control system for maintaining standing balance at erect and other leaning postures and (2) compare the experimental performance of the closed-loop feedback control system to open-loop baseline stimulation for maintaining standing balance at leaning postures in the presence of externally applied perturbations.

## Methods

### Subjects

Two subjects (labeled S1 and S2) with complete SCIs, one a male and one a female participated in the study. The anthropometric and injury characteristics of each subject are shown in Table 1. Both subjects had sensory incomplete/motor complete (AIS B) neurological deficits at mid-thoracic levels (T5/T6 and T4, respectively), and have been successfully using their system for standing and exercise for at least 5 years each. Both subjects signed the consent form approved by the local institutional review board before any experiments were conducted.

**Table 1:** Anthropometric and injury characteristics of the two SCI subjects

Subject ID	Weight (kg)	Weight (N)	Height (cm)	Age	Level	AIS
SI	68.6	673	167.6	61	T5/T6	B
S2	63.5	750	216.0	60	T4	B

Subject S1 was the recipient of a 16-channel [8] implanted neural prosthesis targeting, bilaterally, hip extensors (Gluteus Maximus, GX, Semimembranosus, SM and Adductor Magnus, AM), hip abductors (Gluteus Medius, GM), knee extensors (Vastus Lateralis, VL), ankle plantar/and dorsiflexors (Lateral Gastrocnemius, GS and Tibialis Anterior, TA) and trunk extensors (Lumbar Erector Spinae, LE). All implanted muscles were activated via intramuscular electrodes connected to an implanted pulse generator delivering biphasic charge balanced stimulating waveforms at a frequency of 20 Hz with constant amplitude, taking a value in the range 0–20 mA and variable pulse duration between 0 and 250 µs. She received her implanted system almost 9 years prior to participating in this study and is a regular home user of the neural prosthesis for both exercise and standing function. For these experiments, her implanted system was augmented with surface stimulation electrodes to maximally recruit the hip flexors (Rectus Femoris, RF), back extensors (Thoracic Erector Spinae, TE) and hip adductors (Adductor Brevis, AB) bilaterally. RF was recruited because she has no hip flexor in her implanted system, while TE and AB were recruited because the equivalent implanted counterparts (LE and AM, respectively) were almost fully required for posture maintenance. Surface stimulation was delivered at a constant frequency of 20 Hz, with a fixed 100 mA pulse amplitude, and variable pulse-width (PW) up to 250 µs.

Subject S2 received an implanted neural prosthesis for standing and stepping consisting of two pulse generators: one with 8-channels [10] and the second with 16 channels [8] for a total of 24 channels of stimulation. The system targeted hip extensors (GX, SM and AM), hip abductors (GM), knee extensors (VL), knee flexors (Tensor Fasciae Latae, TF), hip flexors (Iliopsoas, IP), ankle plantar/and dorsiflexors (GS and TA), trunk extensors (LE) and lateral benders (Quadratus Lumborum, QL). One implanted electrode targeted a muscle to assist with knee and hip flexion (Sartorius, SA) on the right side only, and another targeted additional muscle fibers for hip extension (SM) on the left side only. For this subject, three of the quadriceps muscles (the Vasti, VA consisting of VL, Vastus Intermedius, VI and Vastus Medialis, VM) on each side were activated simultaneously via a spiral nerve cuff electrode around the femoral nerve in each proximal thigh well distal to the inguinal ligament to avoid RF which could compromise erect stance by flexing the hip. Similarly, the ankle plantar/dorsiflexors (GS, TA) were activated via spiral nerve cuffs [13] installed below the knee on the tibial and fibular nerves, respectively. All other muscles were activated via intramuscular electrodes [32]. Amplitudes of stimulating currents to the nerve cuff electrodes were limited to constant values between 1 and 2 mA to stay within safe charge densities, while waveform amplitudes to the intramuscular electrodes were assigned constant values between 0 and 20 mA. Stimulus frequency to all electrodes was fixed at 20 Hz, and pulse-widths varied between 0 and 250 µs. Subject S2 received his implanted neural prosthesis 5 years prior to participating in the study, and has been a regular home user of the system for standing and exercise.

### Functional neuromuscular stimulation

For each subject, the targeted muscles were partitioned into two groups. The first consisted of the ‘postural’ muscles (GX, SM, AM, VL and LE for subject S1 and GX, SM, VA and LE for subject S2) which were essential for maintaining an erect standing posture for each of these particular subjects. The stimulation levels (PWs) to these muscles were kept at the baseline values determined clinically for each subject to provide optimal erect standing with minimal bodyweight placed on the UEs for support, and were never modulated during experimental trials. The second group consisted of the ‘controlled’ muscles which were not essential for erect static standing, but which were important in maintaining a posture away from erect or reacting to balance perturbations [31]. The main controlled muscles were hip ab/adductors (GM, AM, AB) and ankle plantar/dorsiflexors (GS, TA). The former were recruited to control motion in the coronal plane while the later, along with some hip flexors (RF) and back extensors (TE), were recruited to control motion in the sagittal plane. Table 2 gives a complete listing for all muscles as well as their functional grouping. It should be noted from this table that a critical muscle for postural control in the ML plane such as the GM was not functional on the left side for S2. Similarly, TA and LE, which would be important for control of posture in the AP plane and AM in the ML plane, were not available for control in Subject S1. These were considered postural muscles since they were required for support, rather than balance, in order to keep her in an erect standing posture (i.e., prevent the knees, hips and trunk from collapse), and thus were unavailable for modulation without increasing the risk of instabilities. These were compensated by adding surface stimulation to RF and TE to help resist forward–backward perturbations and Adductor Brevis (AB) to help resist left–right perturbations. Also from the table, it is noted that both postural and control muscles may contribute to the baseline open-loop stimulation condition.

**Table 2:** List of muscles for subjects S1 and S2 and their function

Muscle	S1				S2			
	Function	$p_{\text{norm}}^{i}$ (μs)	$p_{ \hbox{max} }^{i}$ (μs)	Amplitude (mA)	Function	$p_{\text{norm}}^{i}$ (μs)	$p_{ \hbox{max} }^{i}$ (μs)	Amplitude (mA)
Eight Tibialis Anterior (TA)	P	250	250	20	C (AP)	7	52	12.5
Right Adductor Magnus (AM)	P	250	250	20	C (ML)	0	250	20
Right Vasti (VL or VL + VI + VM)	P	250	250	10	P	24		12.5
Right Lumbar Erector Spinae (LE)	P	73	90	10	C (AP)	0	75	2
Right Gluteus Maximus (GX)	P	170	170	20	P	250	250	20
Right Semimembranosus (SM)	P	250	250	20	P	250	250	20
Right Iliopsoas (TP)					P	0	20	14
Right Tensor Fasciae Latae (TF)					P	250	250	20
Right Sartorius (SA)					P	0	250	20
Right Quadralus Lumborum (QL)					P	0	34	2
Left Tibialis Anterior (TA)	P	17	26	20	C (AP)	27	250	7.5
Left Adductor Magnus (AM)	P	250	250	20	CO (ML)	1	250	20
Left Vasti (VL or VL + VI + VM)	P	130	130	10	P	100	100	10
Left Lumbar Erector Spinae (LE)	P	100	100	10	C (AP)	0	112	6
Left Gluteus Maximus (GX)	P	250	250	20	P,P	250,250	250,250	20,20
Left Semimembranosus (SM)	P	250	250	20	P,P	250,70	250,70	20,20
Left Iliopsoas (IP)					P	0	83	14
Left Quadralus Lumborum (QL)					P	0	23	6
Right Gastrocnemius (GS)	C (AP)	0	36 [8]	20	C (AP)	37 [65]	85	7.5
Right Gluteus Medius (GM)	C (ML)	0	250	20	C (ML)	0	250	20
Right Rectus Femoris (RE) (surface)	C (AP)	0	250	100				
Right Adductor Brevis (AB) (surface)	C (ML)	0	250	100				
Right Thoracic Erector Spinae (TE) (surface)	C (AP)	0	250	100				
Left Gastrocnemius (GS)	C (AP)	0	100 [22]	20	C (AP)	4 [34]	52	12.5
Left Gluteus Medius (GM)	CO (ML)	0	250	20	C (ML)	0	0	0
Left Adductor Brevis (AB) (surface)	CO (ML)	0	250	100				
Left Rectus Femoris (RE) (surface)	C (AP)	0	250	100				

A ‘P’ indicates muscle was always recruited as a postural muscle while ‘C’ implies muscle was recruited as a controlled muscle. Values in square brackets indicate values used during leaning postures. Entries in parenthesis indicate primary plane of action for controlled muscles—AP, anterior–posterior and ML, medial–lateral. An empty entry in the | function implies the muscle was not recruited for that subject

### Feedback signal

While joint angle and CoMA had been successfully utilized as feedback signals for control of standing balance, the former required extensive instrumentation to control multiple joints simultaneously without reducing the degrees of freedom via external bracing [27], while the latter required extensive off-line simulations and human-in-the-loop experimental tuning [2, 30]. For practical purposes in laboratory-based design and exploration studies, more easily acquired signals such as center of pressure (CoP) may be equally useful for gaining insight into the feasibility of various controller structures. The feedback controller described here was based on using overall body CoP as the feedback signal for control of standing balance with FNS. CoP was computed by combining force and moment readings in real time from individual biomechanics platforms (Advance Mechanical Technology Inc, Watertown MA) under each foot during experimental sessions [4]. The AP and ML CoPs under the right (R) and left (L) feet were computed using:

1 $${\text{CoP}}_{\text{AP}}^{\text{R/L}} = - \frac{{{\text{M}}_{\text{ML}}^{\text{R/L}} }}{{{\text{F}}_{\text{IS}}^{\text{R/L}} }}\quad {\text{CoP}}_{\text{ML}}^{\text{R/L}} = - \frac{{{\text{M}}_{\text{AP}}^{\text{R/L}} }}{{{\text{F}}_{\text{IS}}^{\text{R/L}} }}$$

The resultant, whole body CoP, was computed from the formula:

2 $${\text{CoP}}_{\text{AP/ML}} = {\text{CoP}}_{\text{AP/ML}}^{\text{R}} \frac{{{\text{F}}_{\text{IS}}^{\text{R}} }}{{{\text{F}}_{\text{IS}}^{\text{R}} + {\text{F}}_{\text{IS}}^{\text{L}} }} + {\text{CoP}}_{\text{AP/ML}}^{\text{L}} \frac{{{\text{F}}_{\text{IS}}^{\text{L}} }}{{{\text{F}}_{\text{IS}}^{\text{R}} + {\text{F}}_{\text{IS}}^{\text{L}} }}$$

Here, ${\text{M}}_{\text{AP/ML}}^{\text{R/L}}$ is the moment at the right/left foot measured about the AP/ML axis of the force platform and ${\text{F}}_{\text{IS}}^{\text{R/L}}$ is the inferior–superior (vertical) component of the ground reaction force under the right/left foot. The 3 quantities ${\text{M}}_{\text{AP}}$, ${\text{M}}_{\text{ML}}$, and ${\text{F}}_{\text{IS}}$ under each foot were measured by the force platform on which the foot rested.

### Controller design

**Fig. 1:**
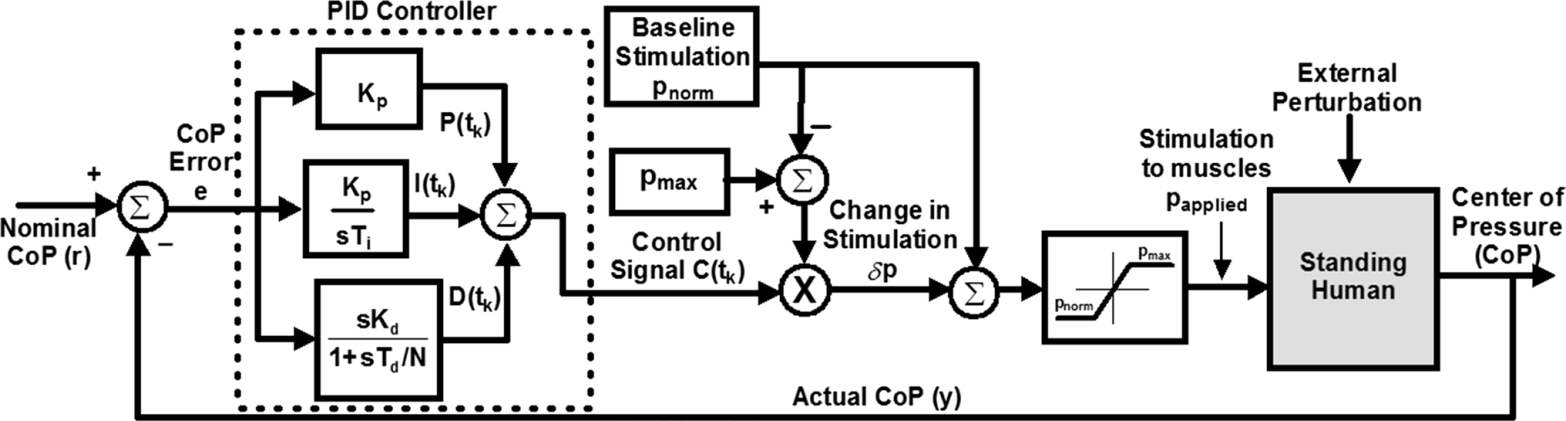
Flow diagram for the feedback control of standing posture. The error in CoP measured by two force platforms on which the subject stood provides input to a PID controller which produces a normalized control signal that was converted to PW values to be applied to the muscles of the hips and ankles. All PWs to be applied to the muscles are limited so as not to go below the nominal value for the muscle and not above the maximum value. s is the Laplace transform parameter

The controller implemented and tested in this study was a modification of the 2-stage controller defined in [1]. The first stage consisted of a PID controller with an output signal that was a function of the error between the nominal CoP of the subject in a given leaning pose and the current CoP during a perturbation. The PID controller, described in Fig. 1, was defined by the following equations in digitized form at time t_k_ [5]:

3 $$C(t_{k} ) = P(t_{k} ) + D(t_{k} ) + I(t_{k} )$$

4 $$e(t_{k} ) = r(t_{k} ) - y(t_{k} )$$

5 $$P(t_{k} ) = K_{p} [r(t_{k} ) - y(t_{k} )]$$

6 $$D(t_{k} ) = \frac{{T_{D} }}{{T_{D} + N\Delta t}}[D(t_{k - 1} ) - K_{p} \cdot N \cdot (y(t_{k} ) - y(t_{k - 1} ))]$$

7 $$I(t_{k} ) = I(t_{k - 1} ) + \frac{{K_{p} \Delta t}}{{T_{I} }}e(t_{k} )$$

In these equations, *C* was the controller output; *P*, *D* and *I* were the proportional, derivative and integral terms. $K_{P}$, $K_{D}$ and $K_{I}$ were the proportional, derivative and integral gains, respectively. $T_{D}$ was the derivative filter time constant, $T_{I}$ the integral time constant, with $T_{D} = K_{D} /K_{P}$ and $T_{I} = K_{P} /K_{I}$; *N* was the derivative filter constant (2 ≤ *N* ≤ 20) and Δt was the sampling period. *e*(*t*) was the error between the nominal (*r*) and actual (*y*) CoP in the AP or ML direction. Although there were separate controllers for AP and ML planes, the two acted simultaneously to correct any changes that occurred in the resultant CoP.

For FNS application, the output $C(t_{k} )$ from the controller as defined by Eq. (3) was used in a relationship that computed the PW to synergistic control muscles as a function of baseline PW and maximum PW as:

8 $$p_{\text{applied}}^{i} = p_{\text{norm}}^{i} + \delta p = p_{\text{norm}}^{i} + C(t_{k} )\left( {p_{ \hbox{max} }^{i} - p_{\text{norm}}^{i} } \right)$$

In Eq. (8), $p_{\text{applied}}^{i}$ was the PW setting for the ith muscle, $p_{\text{norm}}^{i}$, representing the second stage, was the baseline PW applied to the ith muscle earlier determined as the optimum values that allowed for a stable static posture. Thus, it represented a kind of feedforward control mechanism [21]. $p_{ \hbox{max} }^{i}$ was the maximum PW above which muscle force production did not increase. These limiting values had been determined heuristically in trial-and-error experiments for each subject and were pre-programmed into their implant control units. A Simulink block was created in the xPC-Target Real-Time data capture computer (The Mathworks, Natick, MA) to implement the control law in Eq. (8). The output (resulting PW values) from the controller was sent through a cable to the external control unit of the user’s implanted and/or surface stimulation system.

### Controller tuning

Estimates for values of the controller parameters $K_{P}$, $K_{D}$ and $K_{I}$ in Eqs. (3)–(7) were obtained through a series of simulations (in silico *experiments*) with a subject-specific musculoskeletal model of each subject derived from a generic representation of a nominal human (78 kg mass and 178 cm tall). The generic model was a full three-dimensional doubly supported inverted pendulum model of human stance that included Hill-type muscle and tendon actuator dynamics [6]. It was developed, and all simulations conducted, in the Software for Interactive Musculoskeletal Modeling, SIMM (Musculographics, Inc., Santa Rosa, CA). The model was customized to represent subject-specific characteristics by scaling the mass and geometry of individual segments of the body according to regression equations defined in the US Army Anthropometric Survey [24] with subject mass and height as input variables. The muscle force-generating properties were further adjusted to match the isometric forces produced by maximally activating each muscle and measuring the resulting output torque with a computerized robotic dynamometer (Biodex Medical Systems, Shirley NY). Only the stimulated muscles were included in the simulations for an individual with SCI. An impedance controller with time-delays [20] and exponential muscle force scaling [36] based on the known dynamics of human operators was applied at each shoulder of the model to represent voluntary interactions with an assistive device such as a walker. The controller gains and time-delays of this UE model were optimized to match laboratory measurements of the loads exerted on an instrumented walker in response to systematically applied disturbances [33].

In the simulation experiments [7], the model was placed at various postures including erect, forward, forward-right and forward-left [31]. For all leaning postures, values for $p_{\text{norm}}^{i}$ for some muscles were adjusted based on simulation results as described in [6]. The ratios $p_{\text{norm}}^{i} /p_{ \hbox{max} }^{i}$ were used as the muscle activations required to keep the body in static equilibrium at an erect posture and were used for the other postures as well. At each posture, force pulses in varying directions and of varying magnitude from 3 to 20% BW were applied on various parts of the body such as the trunk, pelvis and thighs. A genetic optimization algorithm library [3] was used to find the values of the controller gains with the objective to minimize the forces exerted on the UEs. The particular genetic optimization algorithm used was Asynchronous Parallel Pattern Search Package—APPSPACK (In DAKOTA v6.0, Sandia National Laboratories, Albuquerque, NM). This algorithm searches for optimal values of parameters by doing a search of the optimum point around any selected initial point. The next iteration starts from that local optimum point and so on until a global optimum is obtained. One main advantage of APPSPACK is its ability to solve optimization problems using parallel function evaluations which could be executed on a multi-processor computer for faster convergence to a solution. The parameters obtained from the optimization were used as the initial values for each subject and were further refined with the subject in the loop in preliminary experiments prior to collecting data to closely satisfy subject comfort.

### Experimental protocol

**Fig. 2:**
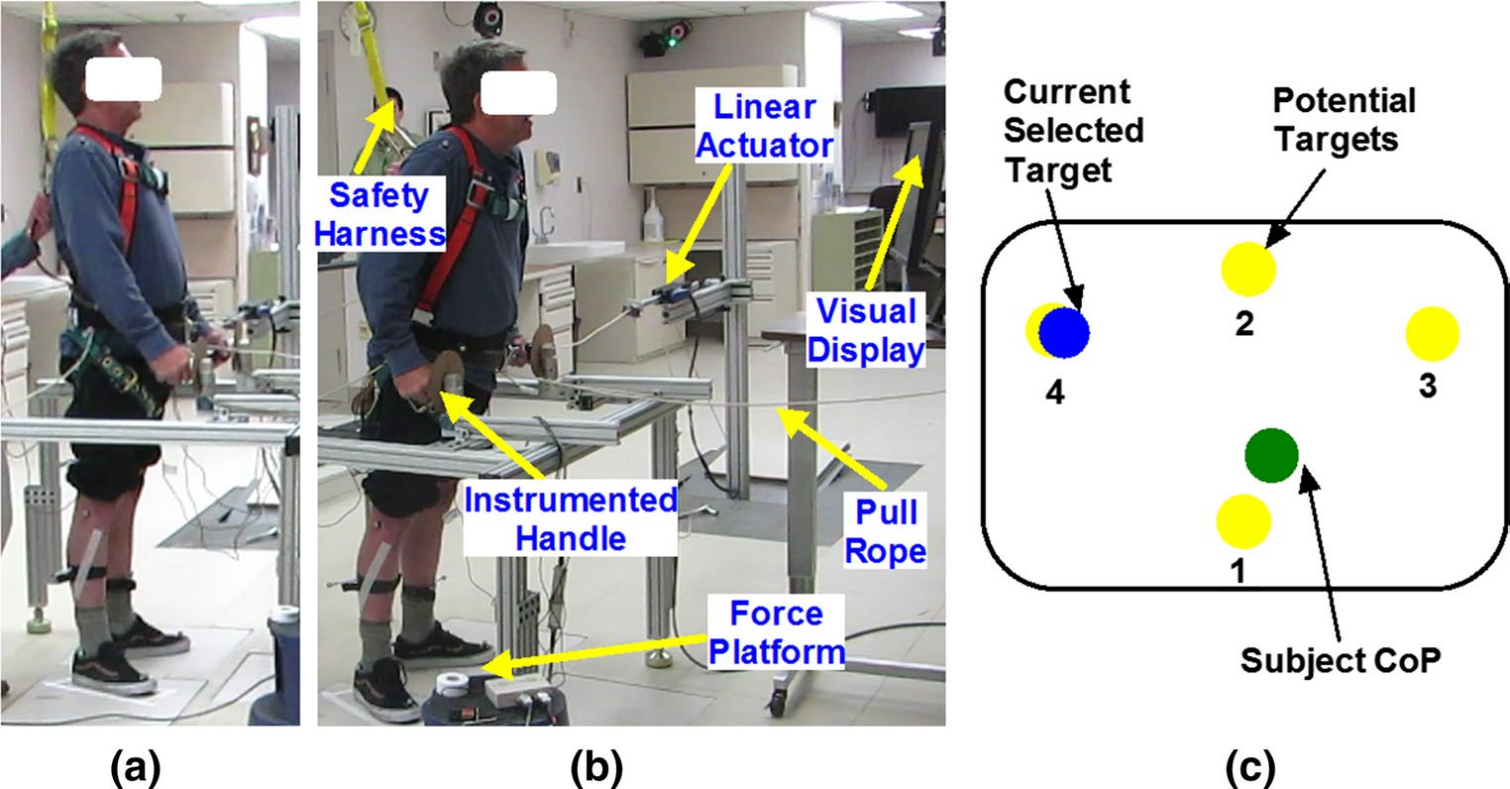
Experimental setup with subject being pulled in 4 directions by linear actuators while **a** standing erect and **b** leaning forward. **c** Visual display showing CoP end-points within the BoS (*black border*). The *yellow circles* represent the optimal target locations where the subject could lean so that their CoP was located there. The *blue circle* represents the current choice for the subject to lean prior to application of perturbations (shown above slightly offset from the *yellow target circle* for forward-left desired lean position). The *green circle* traces the current exact location of the subject’s CoP. At a desired nominal stance, the *green circle* should overlap the *blue circle* (color figure online)

In these experiments, the subjects stood with each foot on a separate force platform and maintained balance by applying corrective loads with their UEs on a walker with handles instrumented with 6-DOF (degree of freedom) load cells (MCW-500, Advanced Mechanical Technology Inc., Watertown, MA). Throughout the experiments, the subjects wore a standard fall prevention rock-climbing harness connected to a structurally reinforced overhead hook via a safety lanyard (Guardian Fall Protection Inc., Kent WA) meeting specifications set by the Occupational Safety and Health Administration, and were spotted by a study staff at all times. The locations of the subject’s feet on the force platforms were demarcated with cloth tape to allow for repeatability of foot placement in subsequent trials. A typical experimental setup is depicted in Fig. 2a, b.

#### Determination of CoP limits

Prior to each experimental session, the limits of CoP that the subject could comfortably traverse and maintain while leaning in the erect, forward and diagonal directions (forward-right and forward-left) were determined. The CoP locations for the 4 postures defined the placement of four yellow circles in a visual display placed in front of the subject throughout the rest of the experiments. In the display, depicted in Fig. 2c, a moving green circle represented the current CoP location of the subject and a static blue circle represented the target nominal static CoP for the posture. In each trial, subjects were asked to shift their bodies so as to ensure the blue and green circles overlapped and remained coincident prior to application of each perturbation. Throughout the experiments, the knees and hips were kept in full extension by continuous constant stimulation of the knee and hip extensor muscles which belonged to the postural muscle sets.

#### Application of perturbations

In each experimental session, external perturbations were applied in random order in the forward (FO), right (RI), backward (BA) and left (LE) directions. Controlled and repeatable force perturbations were generated by four electromagnetic linear actuators (STA2506, Copley Controls, Inc., Canton, MA) placed orthogonal (front, right, back and left) to the standing subject. The actuators were mounted on specially designed aluminum support structures (80/20^®^, Inc., Columbia City, IN) rigidly fixed to either the wall or the laboratory sub-flooring. Consecutive operation of the actuators was conducted through a custom programmed block developed in Simulink-xPC-Target (the Mathworks, Natick, MA) real-time interface. All forces were applied close to the center of mass of each subject via ropes from the actuators to a weight-lifting belt worn around the waist.

Distinct perturbation magnitudes were identified for each subject in each of the four directions. The maximum magnitude in each direction was determined based on subject comfort but was also just large enough to ensure efficient recovery to a quiet stance after the perturbation. The perturbation magnitude levels in the FO, RI, BA and LE directions for S1 were 3, 3, 2, and 3% BW and for S2 were 6, 6, 4 and 5% BW, respectively. Each force pull was a trapezoid pulse that rose from zero to maximum in 100 ms, dwelled for 500 ms and returned to zero in 100 ms. In each trial, the perturbations were spaced 5 s apart to enable the subject resume a stable quiet stance as defined by overlap of the green and blue circles in Fig. 2c.

### Data collection

In each trial, the subject was perturbed 3 times along each of the 4 ordinal directions for a total of 12 cycles per trial with the perturbations applied in random order in each trial. Test trials were conducted prior to commencing data collection to familiarize the subject with the perturbations and the overall protocol. A typical trial lasted about 3 min from standing up with open-loop baseline stimulus pattern to sitting back in the subject’s own wheelchair. Trials were conducted with either the open-loop baseline stimulation for that posture or with the feedback controller active in random order. A rest period of 5–10 min was allowed between trials to enable the adequate muscle recovery and minimize fatigue effects. A total of 6 trials were collected with the subject in each of the 4 postures (erect, forward, forward-right and forward-left). This resulted in a total of 9 cycles for each of the 32 conditions (4 leaning directions, 4 force perturbation directions and 2 controller conditions).

Force plate data (CoP), walker handle forces, muscle stimulus levels and actuator pulls were all captured as analog data via the Simulink-xPC-target model of the control system using a data acquisition card, PCI-6071E AD (National Instruments Inc., Austin, TX). All data were sampled at 40 Hz.

### Outcome measures

The following outcome parameters were computed to assess the effect of feedback control over baseline stimulation: (a) normalized maximum resultant UE force and two controller performance measures (b) maximum resultant CoP and (c) resultant CoP root-mean-square deviation (RMSD). The maximum resultant UE force and maximum CoP were calculated as the absolute maximum changes over the average of baseline values. The RMSD was calculated as the square root of the mean of the sum of squares of the differences between the values at each frame of data from the baseline average values, which for all parameters were computed for 0.30 s prior to perturbation application. The maximum resultant UE force when the subject was perturbed in a given direction was normalized by dividing the UE force by the perturbation force applied in that direction. All 3 outcome measures were computed on a cycle-by-cycle basis, using data from when perturbation commences to the end of the cycle, and then averaged to determine the mean values.

### Statistical analysis

In view of the small size of the sample data, the Wilcoxon Signed-Rank nonparametric statistical tests were used to compare the outcome measures for the two conditions (a) with open-loop baseline stimulation and (b) with closed-loop feedback controller active. Subjects acted as their own controls for the purpose of testing the difference between the two controller cases. In addition, we also conducted Kruskal–Wallis tests to assess the effects of leaning posture and perturbation direction on the UE forces when the feedback controller was active. Multiple comparisons, using Dunn’s criterion, were later conducted to determine which leaning directions resulted in larger maximum resultant UE force. The significance level was set at 0.05.

## Results

### Controller parameters

The parameters for the feedback PID controller defined in Eqs. (3) to (7) are displayed in Table 3 for each subject. The proportional gains ranged between 1.1e−3 and 15e−3, while the derivative and integral gains varied between 1.1e−3 and 2.3e−3 and 1.2e−3 and 3.2e−3, respectively. Most of the gains were about twice as large for S2 than for S1; the only exception being the AP proportional gain which was almost 3 times larger for S1. The final values were decided based on user comfort with the changes in stimulation by the feedback controller in the presence of CoP changes. The limiting values $p_{\text{norm}}^{i}$ and $p_{ \hbox{max} }^{i}$ for the muscles recruited are listed in Table 2 for the two subjects. From Table 2, it is evident that the nominal or baseline stimulation PWs were close to the maximum values for most of the postural muscles (labeled ‘P’). In addition, the controlled muscles group (labeled ‘C’) differed between the subjects depending on their postural demands.

**Table 3:** PID feedback controller gains for the two SCI subjects

Subject ID	*K*_*P*_ (×l.0e^−3^)		*K*_*D*_ (×l.0e^−3^)		*K*_*I*_ (×l.0e^−3^)		*N*
	AP	ML	AP	ML	AP	ML	
SI	15.0	1.1	1.2	1.1	1.2	2.5	8
S2	4.0	2.5	2.2	2.3	1.5	3.2	8

*K*_*P*_, *K*_*D*_ and *K*_*I*_ are the proportional, derivative and integral gains, respectively, for each of the two planes (anterior–posterior, AP and medial–lateral. ML). *N* is the derivative filter constant

### Typical responses

**Fig. 3:**
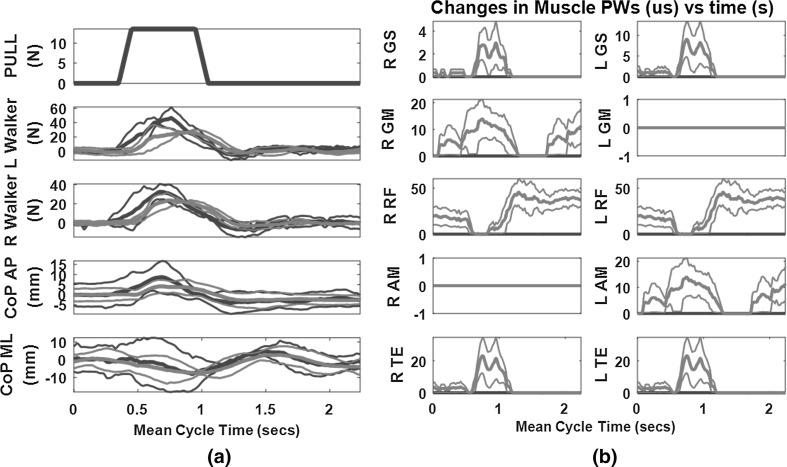
Sample plot of the results of averaging several cycles of a forward perturbation for subject S1 standing in an erect posture. **a** Pull force profile along with walker (UE) forces exerted and resulting changes in CoP in the AP and ML directions; **b** resulting changes in muscle pulse-widths (μs) for the controlled muscles for this subject—R GS, Right Gastrocnemius; L GS, Left Gastrocnemius; R GM, Right Gluteus Medius; L GM, Left Gluteus Medius; R RF, Right Rectus Femoris; L RF, Left Rectus Femoris; R AM, Right Adductor Magnus; L AM, Left Adductor Magnus; R TE, Right Thoracic Erector Spinae; L TE, Left Thoracic Erector Spinae. Except for the pull force profile [top plot in (**a**)] *blue color* represents baseline (open-loop) stimulation quantities while *red color* represents feedback controller quantities (color figure online)

Figure 3 shows typical plots of the perturbation force applied and the resulting change in CoP, UE forces and muscle PWs for one of the subjects (S1). Except for the perturbation force, the other traces show the means ±1 standard deviation over the 9 cycles of data captured for the given condition. For S1/S2, each cycle of a trial lasted approximately 2.2/2.7 s and consisted of a period of quiet standing 0.35/0.5 s prior to application of a perturbation and an additional 1.2/1.5 s after the applied force returned to zero.

The changes in PW values for the controlled muscles as the CoP changed as a result of perturbation in the FO direction are also shown in Fig. 3. It is clear that muscles from both planes were recruited simultaneously to correct for non-orthogonal movement of the CoP, regardless of whether the perturbation was applied in a purely cardinal plane (AP or ML). For the FO perturbation illustrated, right and left GS and TE were recruited to resist the positive increase in AP CoP. During the period when the AP CoP was at a peak, the subject might have shifted in the rightward direction (negative ML CoP) thus causing right GM and left AM to activate simultaneously. There was not enough change in positive ML CoP to cause the left GM and right AM to activate thus the two muscles remained silent throughout the maneuver.

### Outcome measures

**Fig. 4:**
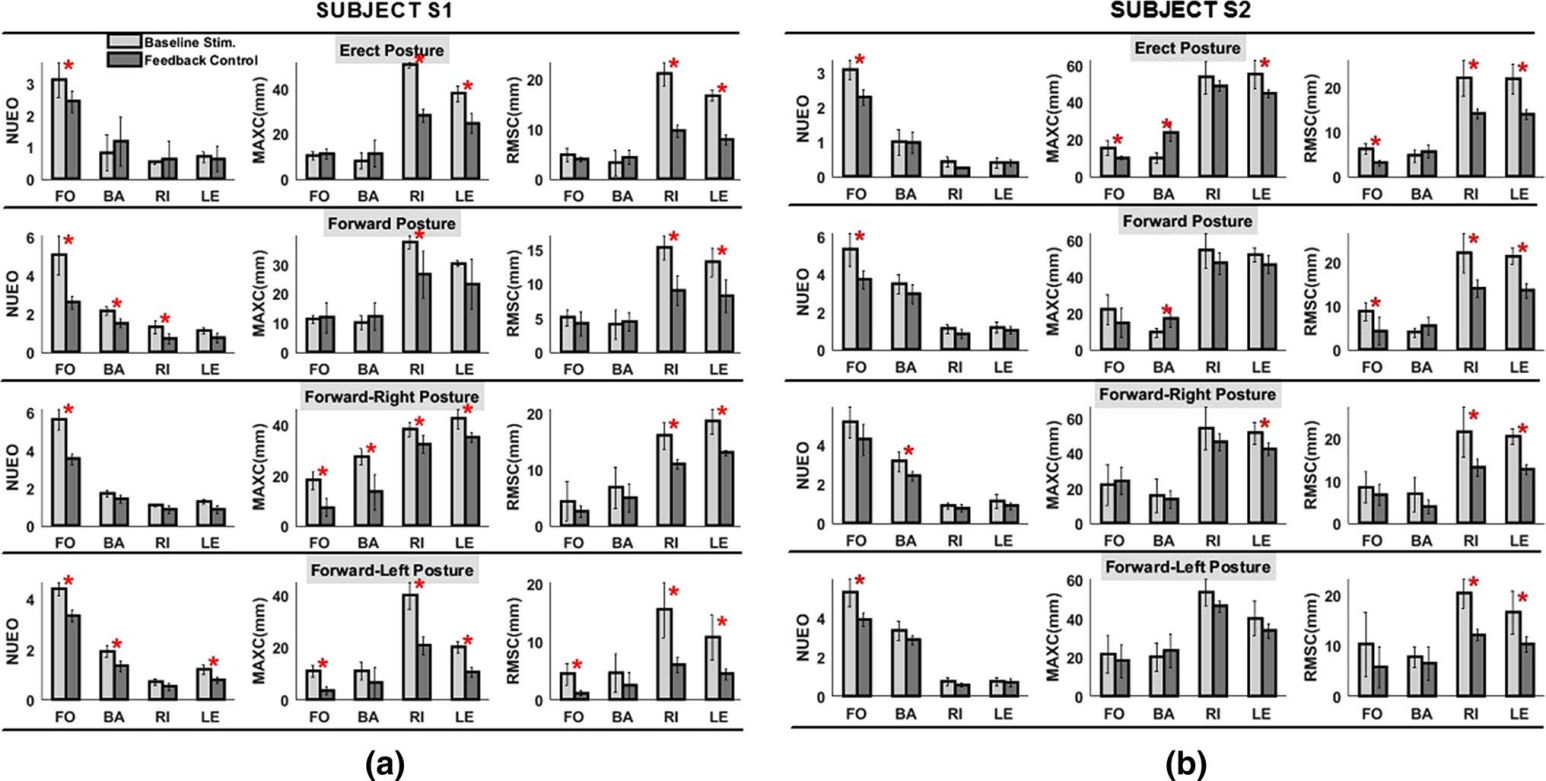
Outcome parameters for **a** Subject S1 and **b** Subject S2 during perturbations in 4 directions (forward, FO, backward, BA, rightward, RI and leftward, LE) for the four leaning postures—erect, forward, forward-right and forward-left. The *bar graphs* show the mean values for the 3 outcome variables with *error bars* representing ±1 standard deviations. The *light shaded bars* represent the mean values with baseline stimulation, while the *darker shaded bars* represent the mean values with feedback controller. An *asterisk* on top of a pair of bars implies statistically significant differences between the median value with baseline stimulation and that with feedback controller at *p* < 0.05. *NUEO* normalized UE overshoot, *MAXC* maximum CoP change and *RMSC* root-mean-square deviation in CoP

Figure 4 shows the mean values (±1 standard deviation) of the 3 outcome measures for the two subjects when standing in each of the 4 postures (1) erect, (2) forward, (3) forward-right and (4) forward-left. Each sub-plot contains the bar graphs for a single outcome measure when the subject was perturbed in each of the 4 directions—forward (FO), backward (BA), rightward (RI) and leftward (LE), respectively. The lighter bars represent the values when open-loop baseline stimulation was active, while the darker bars represent the values when the feedback controller was active. An asterisk on top of a pair of bars indicates that the value for the outcome measure with feedback controller was statistically significantly different from the value with baseline stimulation at *p* < 0.05. Percentage changes in the various parameters (100*(baseline stimulation − feedback controller)/baseline stimulation)) are also presented in Table 4. Highlighted numbers in the table are positive changes indicating a higher value resulting with open-loop (baseline) stimulation compared to feedback control.

**Table 4:** Percent change in three outcome measures as function of posture (ER, FW, FR and FL) and perturbation direction (FO, BA, RI and LE) for the two subjects S1 and S2

Posture	Parameter	Perturbation direction and subject							
		Forward (FO)		Backward (BA)		Right (RI)		Left (LE)	
		S1	S2	S1	S2	S1	S2	S1	S2
Erect (ER)	Maximum resultant UE force	**22.1**	**25.7**	−43.2	**1.7**	−19.4	**42.7**	**9.2**	**1.8**
	Maximum resultant CoP	−5.9	**35.2**	−38.2	−135.6	**44.2**	**8.6**	**34.9**	**18.7**
	RMSD in CoP	**18.1**	**52.3**	−37.3	−21.7	**54.2**	**36.4**	**52.7**	**36.5**
Forward (FW)	Maximum resultant UE force	**48.8**	**30.3**	**30.8**	**15.8**	**46.5**	**22.5**	**32.7**	**12.1**
	Maximum resultant CoP	−5.4	**33.8**	−20.9	−83.1	**29.4**	**12.8**	**23.1**	**10.0**
	RMSD in CoP	**17.0**	**50.8**	−9.1	−38.3	**41.1**	**36.8**	**37.3**	**36.9**
Forward right (FR)	Maximum resultant UE force	**37.3**	**16.9**	**17.8**	**23.6**	**21.9**	**13.1**	**32.0**	**22.1**
	Maximum resultant CoP	**60.2**	−9.5	**51.5**	**14.8**	**15.2**	**14.4**	**17.4**	**17.5**
	RMSD in CoP	**39.8**	**21.1**	**26.7**	**43.5**	**31.2**	**39.2**	**30.0**	**37.4**
Forward left (FL)	Maximum resultant UE force	**24.0**	**26.0**	**29.7**	**14.8**	**27.8**	**24.9**	**35.1**	**5.5**
	Maximum resultant CoP	**69.1**	**16.5**	**42.8**	−16.0	**48.0**	**13.5**	**47.7**	**16.3**
	RMSD in CoP	**74.4**	**44.3**	**49.0**	**16.7**	**61.2**	**40.4**	**59.2**	**37.7**

Bold values are positive changes indicating a higher value resulting with open-loop (baseline) stimulation compared to feedback control

#### Maximum resultant UE force

As evident from Fig. 4, the maximum resultant UE force for both subjects was generally larger when the subject was perturbed in the FO and BA directions; especially so far perturbations in the FO direction. This may be because a forward perturbation tended to increase the transient UE load further by momentum transfer to the support device handles. In general for all FO perturbations the feedback controller resulted in significantly lower values of maximum resultant UE force for all postures in both subjects. The reductions in resultant UE force with feedback controller varied between 20 and 50% for S1 and between 2 and 42% for S2 as shown in Table 4. Kruskal–Wallis analysis indicated that leaning direction did not have any significant effect on feedback controller reduction in resultant UE forces irrespective of perturbation direction for both subjects (*p* < 0.05). Similar analysis also indicated that the reductions in UE force were not affected by perturbation direction irrespective of posture for both subjects (*p* < 0.05).

#### Controller performance measures

Unlike the maximum resultant UE force, the maximum resultant CoP was generally larger for perturbations in the RI and LE directions than in the FO and BA directions as shown by the subplots in the middle columns in Fig. 4. The feedback controller resulted in significantly larger reductions of this parameter for both subjects (Fig. 4). However, there was no statistically significant difference between feedback controller and baseline stimulation in the maximum CoP when both subjects were perturbed in the BA direction. For both subjects, feedback controller tended to increase this parameter during the erect and forward stances as seen in Table 3 (negative values in BA columns).

From the third columns in Fig. 4, the RMSD followed similar patterns as the maximum resultant CoP with larger values occurring for perturbations in the RI and LE directions than in the FO and BA directions. For both subjects, the feedback controller resulted in significantly lower RMSD compared to baseline stimulation for most perturbations in the RI and LE directions. The values for the two controllers remained about the same for most of the perturbations in the FO and BA directions.

## Discussion

Recent interests in standing balance controllers seek to expand the capability to postures that are away from erect such as those that could be made in attempts to acquire objects from shelves, racks, etc. while standing. Stability at such leaning postures is important to ensure safety of FNS users. The current study is the first to examine the potential to expand the effect of feedback control systems to postures that are away from erect by perturbing the subjects in different directions with external forces of different magnitudes, while they were at these leaning postures. A global postural variable in the form of the center of pressure (CoP) was used as a feedback signal for control. Cycle to cycle and trial repeatability were guaranteed, first by ensuring that foot placement remained at the same location on the force platforms throughout the experiments. Secondly, visual feedback was employed to ensure that the starting point (CoP location) for all perturbation cycles remained consistent between trials and test sessions, and repeatability of the disturbances was guaranteed by generating the applied forces with calibrated, computer controlled linear actuators. Finally, in all trials, the order of perturbation directions was completely randomized to ensure that learning effect and bias were minimized. Perturbation force magnitudes (<6%BW) were chosen to ensure that fluctuations of the CoP remained within the BoS and did not evoke other balance strategies such as reactive stepping. Preliminary reports of such control systems where reactive steps were solicited from an SCI subject using FNS for standing when perturbations exceeded nominal limits have also been reported [15].

We examined the effect of the feedback control system by comparing its performance to open-loop baseline stimulation that kept the stimulation levels at values appropriate for the acquired leaning postures. The comparison between the two control systems was carried out on the basis of standard quantitative performance measures. The first outcome measure was maximum resultant UE force. This quantity has been identified as an important measure for standing with FNS [25, 34] mainly because it ultimately determined the ability of the users to be able to use their UEs for activities other than to maintain balance. Maximum resultant CoP is one of the transient response characteristics of a feedback control system, while the RMSD is one of the response attributes [1]. Unlike the maximum CoP which was related to a single frame in the temporal history of the CoP, the RMSD is representative of the complete change that takes place throughout the temporal history of the CoP during a perturbation cycle. In the majority of the outcome measures examined, our study indicated significant benefits of the feedback controller over open-loop baseline stimulation. The main exception where the feedback closed-loop controller did not produce significant change in the outcome measures was in the case of posteriorly directed perturbations especially for the erect and forward-leaning postures for S1 and for all postures for S2. This may be due to the fact that the CoP was located near the posterior of the boundary of the BoS very close to the heels in the erect posture. In Subject S1, TA was used as a postural muscle and hence was not available for feedback control which may have contributed to poor performance in the BA perturbations for that subject.

It was also observed that the maximum resultant CoP and RMSD values were larger in the ML plane than the AP plane, indicating that the system was more compliant in the medial–lateral direction. This may be due to the relative strength of the postural hip/trunk extensor musculature compared to the hip/trunk ab/adductors utilized for control. Also, the UE force exerted to counteract the perturbations was larger in the AP plane than in the ML plane, possibly adding to the stiffness observed in the fore-aft direction.

With respect to significant reductions of the maximum resultant UE force compared to open-loop baseline stimulation, similar observations were made by Abbas and Chizeck [1] in their medial–lateral perturbation experiments with the subject in an erect posture. In their studies, they observed reductions in UE force of up to 13 N in the ML direction and up to 9 N in the inferior–superior direction. Although they attributed these changes to trial-to-trial variations, we believe it was more possibly the effect of their feedback controller that caused the reductions. In our study, the reductions in UE force were too large and consistent to be attributed to trial-to-trial variations. This may be confirmed by the work reported in Nataraj et al. [29] in their AP and ML perturbation experiments with a subject standing in an erect posture. Those results indicated that feedback control of CoMA resulted in reduction of UE force of the order of 26, 8, 33 and 40% based on perturbations in FO, BA, RI and LE directions, respectively. All these were considered significant except for the value for perturbation in the BA direction (8%). These were of similar order of magnitude as maximum values obtained for the same perturbation directions for both subjects in our study (Table 4). AP plane controllers such as those of Gollee et al. [14] were also able to robustly maintain standing balance by varying plantarflexor stimulation. Since the knees, hips and back were locked and the hands were free in their experiments, comparison of their results with those of the current study would be difficult.

Kruskal–Wallis analysis accompanied by post hoc analysis of the results indicated that the effect of the feedback controller in terms of reducing all three outcome measures over baseline stimulation was not affected by the posture attained by any of the two subjects nor by the direction in which the perturbation was applied. This is an indication that potentially a single controller designed to work in AP and ML planes was enough to accommodate different situations for an FNS user standing with a neuroprosthesis. Although it is difficult to make comparison between the subjects, it was observed that the maximum changes in CoP and RMSD for Subject S2 due to RI and LE perturbations were generally larger than those for Subject S1 especially in the forward-leaning postures. This may be because of the loss of output from one of the critical ML controlled muscles, GM for S2 and the presence of additional ML controlled muscles, AB, for S1.

Overall, these results indicate the potential for feedback control to reduce resultant maximum UE force by significant amounts in individuals with SCI using FNS for standing in leaning postures, as well as previously demonstrated for nominal erect postures. It is difficult or impossible at this time to make claims on the clinical significance of the outcomes. Nevertheless, it should be mentioned that a major long-term goal of the work is to enable neuroprostheses users maintain standing balance hands-free from any support device thus allowing them use the arms for activities of daily living. While this is far from being achieved, our focus has been on exploring ways to minimize the UE effort with the goal of minimizing the fatigue that accompanies use of the arms for support. This is perhaps more so in postures that lean away from erect. Thus, any step that achieves a reduction in UE effort will be a step toward that end [11]. In addition, reduction in CoP changes under perturbations is significant in the sense that changes in the CoP that result in it moving outside the BoS could cause potentially injurious falls. This is again perhaps more so in leaning postures where the CoP could potentially approach the edge of the BoS. In such situations even a small change in CoP could spell the difference between safe restoration of posture or a fall. The ability of a feedback controller to reduce the CoP change under perturbations over baseline open-loop stimulation could imply that it has a higher potential to prevent such catastrophes.

There were a number of limitations to the current study that would warrant examination in the future. First, all the synergistic muscles for a given plane acted simultaneously. A more detailed control paradigm should recruit each muscle independently in a synergistic manner. This is a major drawback of using PID control which was not optimal for multi-input multi-output systems. An artificial neural network such as that used in [29] or some other modern control approaches [14] would address this issue at the expense of more extensive periods of tuning. A second limitation is the relatively small number of synergistic muscles in the controller group. Future studies should consider including a larger number of muscles to be recruited via implanted, percutaneous or surface stimulation approaches. Thirdly, all perturbations were conducted with the subjects holding onto a support device with both hands. It should also be interesting to examine single-handed balance as most activities of daily living will involve this scenario. Finally, the results reported were for trials captured in a single session on a single day. Replicating the results with more subjects and repeating the experiments on several days or sessions to determine the effects of these replications on reproducibility of the results will be examined in future studies. While muscle fatigue has been a major issue for FNS systems for several years where FNS technology was based on surface stimulation, its effects have not been a major concern in many subjects with implanted systems; especially where the users continuously exercise and use their systems at home. All the subjects in the current study can stand up and remain standing for more than 30 min at a time; before any signs of fatigue start to show. This is in contrast to what was observed in other past studies [14, 25] where the subjects showed signs of fatigue within 3 min of standing. Another issue of interest is the potential effect of the UE forces on the CoP since both were used as outcome measures. A close examination of Fig. 3a shows that whereas with baseline open-loop control the UE forces and CoP values rose to their maxima at about the same time, the CoP tended to lag behind the UE forces when the feedback controller was activated. Thus, there is the potential that the UE force changes could augment the increase in stimulation PW to influence the change in CoP. The extent of this influence and how it could affect the performance of feedback control systems will be the subject of future work.

## Conclusions

We have designed and deployed a real-time feedback controller based on center of pressure for standing balance with FNS after SCI. The controller was tested in two individuals with thoracic level SCI who had been implanted with various FNS systems for standing and stepping. The results showed that the feedback controller reduced the maximum resultant UE force and other controller performance measures, compared to baseline open-loop stimulation, not only when the subjects were standing erect but also when they were leaning in various postures in the forward direction. The larger reduction in maximum resultant UE force indicated the future potential for users of standing balance neuroprostheses to use their UEs for activities of daily living while standing, even in leaning postures. Statistical analysis indicated that feedback controller performance in terms of reducing maximum resultant UE, maximum CoP and RMSD were not affected significantly by the posture attained nor by the direction in which the perturbation was applied; implying that one controller design could be relied upon to provide disturbance rejection for FNS standing neuroprostheses designed to assist with maintaining non-erect postures to enable users prepare to perform ADL tasks that require deviation from the nominally vertical erect posture. The ability to reduce maximum CoP change and RMSD implies that potential falls resulting from the CoP moving outside the BoS as a consequence of disturbances occurring in leaning postures will be reduced.
